# Record-breaking warming and extreme drought in the Amazon rainforest during the course of El Niño 2015–2016

**DOI:** 10.1038/srep33130

**Published:** 2016-09-08

**Authors:** Juan C. Jiménez-Muñoz, Cristian Mattar, Jonathan Barichivich, Andrés Santamaría-Artigas, Ken Takahashi, Yadvinder Malhi, José A. Sobrino, Gerard van der Schrier

**Affiliations:** 1 Global Change Unit, Image Processing Laboratory, University of Valencia, C/Catedrático José Beltrán 2, 46980 Paterna, Valencia, Spain; 2 Laboratory for the Analysis of the Biosphere, Department of Environmental Sciences and Renewable Natural Resources, University of Chile, Av. Santa Rosa, 11315, Santiago, Chile; 3 Instituto de Conservación, Biodiversidad y Territorio, Universidad Austral de Chile, Valdivia, Chile; 4 Center for Climate and Resilience Research, Universidad de Chile, Santiago, Chile; 5 Laboratoire des Sciences du Climat et de l’Environnement, 91191 Gif-sur-Yvette, France; 6 School of Geography, University of Leeds, Leeds LS2 9JT, UK; 7 Department of Geographical Sciences, University of Maryland, College Park, MD 20742, USA; 8 Instituto Geofísico del Perú, Lima, Peru; 9 School of Geography and the Environment, University of Oxford, South Parks Road, Oxford OX13QY, UK; 10 Royal Netherlands Meteorological Institute (KNMI), The Netherlands

## Abstract

The El Niño-Southern Oscillation (ENSO) is the main driver of interannual climate extremes in Amazonia and other tropical regions. The current 2015/2016 EN event was expected to be as strong as the EN of the century in 1997/98, with extreme heat and drought over most of Amazonian rainforests. Here we show that this protracted EN event, combined with the regional warming trend, was associated with unprecedented warming and a larger extent of extreme drought in Amazonia compared to the earlier strong EN events in 1982/83 and 1997/98. Typical EN-like drought conditions were observed only in eastern Amazonia, whilst in western Amazonia there was an unusual wetting. We attribute this wet-dry dipole to the location of the maximum sea surface warming on the Central equatorial Pacific. The impacts of this climate extreme on the rainforest ecosystems remain to be documented and are likely to be different to previous strong EN events.

The Amazon basin has warmed by 0.5 °C since 1980, with stronger warming during the dry season and over the southeast^1^,^2^. Together with warming, the last decade has experienced two major droughts in a very short period^3^,^4^,^5^,^6^, raising concerns about the resilience of tropical forests to extreme droughts and impacts of global warming over this biome. Extreme climatic events over Amazonia are mainly linked to El Niño-Southern Oscillation (ENSO) conditions in the tropical Pacific, but also to sea surface temperature (SST) anomalies in the tropical Atlantic, or to a combination of both^7^. The occurrence of these extreme events severely disrupts the livelihood of riverine populations^7^ as well as the water and carbon cycling of the extensive tropical forests^2^,^8^,^9^,^10^,^11^.

Warm EN events are associated to periodic droughts in Amazonia because of a suppression of the convection and thus rainfall in northern, eastern and western Amazonia^5^,^7^,^12^. Because of the different pattern observed in the tropical Pacific SST anomalies during El Niño events, two types of El Niño have been recently suggested depending on whether the maximum warming is located in the Eastern Pacific (EP) or the Central Pacific (CP). The impacts of these two types of El Niño on the Amazon climate and ecosystems can be markedly different because of the particular convection patterns and atmospheric response linked to EP and CP warmings^13^. These two EN flavours do not imply a dichotomy, as events can be found in-between^14^. In fact, the EN of 1982/83 and 1997/98 can be considered to belong to a separate strong EN regime, with particularly large warming in the EP, as opposed to a moderate regime as occurs for the other observed EN, thus leading to a reinterpretation of the canonical and Modoki El Niño commonly associated to EP and CP anomalies, respectively^15^,^16^.

In particular, the 1997/98 event was considered as the climate event of the twentieth century for its extraordinary magnitude with consequences at global scale^17^. In 2014, anomalous warm SSTs in the central Pacific together with anomalous westerly wind patterns led to some agencies and ENSO experts to alert about the possibility of the first extreme EN event since 1997^17^. Although the EN-2014 conditions were finally not strong enough for this to happen, its warming remnants in the central Pacific may have contributed to start the strong EN-2015^18^. This is the first strong EN in the last 18 years, and it could be expected to result in strong warming and drought over Amazonia, amplified by the ongoing warming trend. Here we use reanalysis and observation-based climate datasets to quantify the warming and drought severity during 2015/2016 over the Amazon forest in the context of the two previous strong EN events (1982/83 and 1997/98).

## Results

### Climate data

We use monthly sea/land surface temperatures and the self-calibrating Palmer Drought Severity Index (scPDSI)^19^ computed using meteorological fields from the ERA-Interim (ERA-I) reanalysis between January 1979 and March 2016. ERA-I provides up-to-date physically consistent meteorological fields and has been shown to perform better than other modern reanalyses in Amazonia^20^. Sea surface temperature anomalies were analyzed over the Pacific and Atlantic regions EN12, EN34, TNA and TSA (Supplementary Fig. S1). Additionally, we used the E and C indices to assess the contribution of the EP and CP types of El Niño^15^.

Land surface temperature (LST) anomalies and scPDSI were analyzed over the Amazon forest area defined as Evergreen Broadleaf Forest class in MODIS 0.05° resolution MCD12C1 land cover product (Supplementary Fig. S1). Details on the climate and remote sensing datasets, as well as processing methods, are provided in the Methods section. Additional results and intercomparison between products are also included in the Supplementary Material.

### Evolution of surface temperature anomalies

SST anomalies over EN34 region show a peak >2.5 °C in November 2015, representing the highest anomaly since 1979, comparable and perhaps slightly higher than the 1997 and 1982 values (Supplementary Fig. S2), although the global warming trend and differences among datasets complicates ranking these events. The temporal evolution of SST anomalies during the developing EN year (January to December) in 1982, 1997 and 2015 are similar, with an increasing trend from January to the peak in November in 1997 and 2015, and the peak in December in 1982, with 2015 typically showing the strongest warming. SST warm anomalies show a decrease to neutral conditions from January 1983, 1998 and 2016 for the three EN events, respectively. SST anomalies over other sensitive regions (e.g. EN12 and the TNA-TSA gradient) are markedly different during the three events (Supplementary Fig. S2), which may explain the different local and regional effects of rainfall patterns over land.

**Figure 1:**
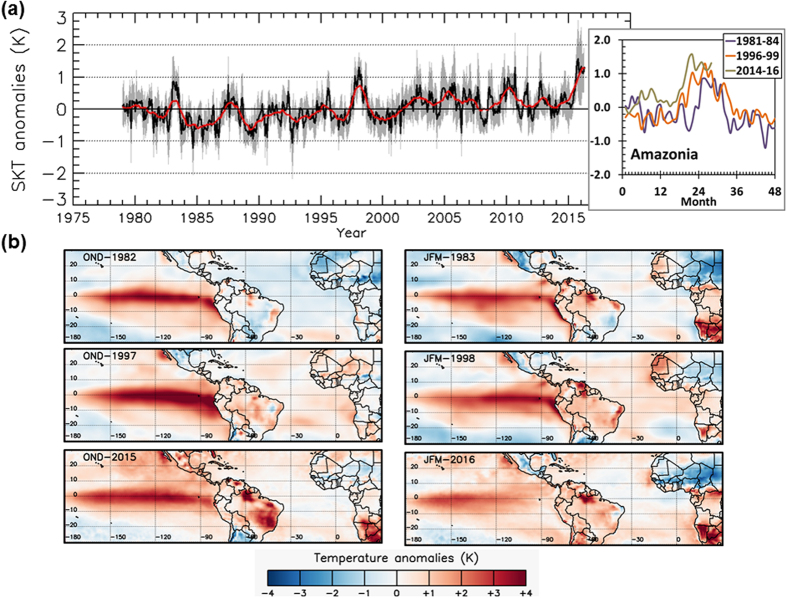
Sea and land surface temperature anomalies during the three strong EN events (1982/83, 1997/98, 2015/16). (**a**) Monthly series of land surface temperature anomalies over Amazonia since 1979, with a running mean for a period of 12 months overlapped. Also shown a comparison of temperature anomalies for the three EN events. (**b**) Spatial patterns of both SST and LST anomalies for the OND and JFM seasons in 1982/83, 1997/98 and 2015/16. Data visualisations produced using IDL v8 (Exelis Visual Information Solutions, Boulder, Colorado).

Land surface temperature anomalies over Amazonia broke a record in 2015 (Fig. 1a). Warming peaked in October 2015, and was stronger by 1.5 °C than the peak observed in October 1997, and 2 °C stronger than the peak observed in January 1983. In fact, the warming over Amazonia is hardly observable during the OND season in 1982. The warming was more clear during the OND season in 1997, but the OND season in 2015 shows the widest and strongest warming (Fig. 1b). Warming remnants are also observed in JFM-1998 and JFM-2016, and a cooling transition in JFM-1983.

However, one of the most significant differences between the three events is observed for the pre-EN years, namely, 1981, 1996 and 2014. Warming over Amazonia is also evident in 2014, with a peak in September 2014 (anomaly higher than 1 °C), whereas cooling or neutral conditions are observed in 1981 and 1996 (Fig. 1a). The warm conditions in 2014 (in both the Amazon basin and the adjacent sea regions) that may helped to the ignition of the EN-2015, which were not observed in 1982 and 1981 and 1996, are clearly evidenced in the spatial distribution of thermal anomalies (Suplementary Figs S3–S5).

### The different faces of El Niño: EP and CP contributions

The contrasting temperature anomalies can be attributed to some extent to the different contribution of the EP and CP SST anomalies among the three EN events. Analysis of E and C indices shows that EN events in 1982 and 1997 are characterized by a strong contribution of the E index (> +3) (Supplementary Fig. S6a). The contribution of the E index to EN-2015 is also strong (about +2), but considerably below the values obtained for EN-1982 and EN-1997. However, EN-2015 is characterized by a strong contribution of the C index (> +2), with a similar C index than the value reached in EN-2009 (Supplementary Fig. S6b). The C contribution was low for EN-1982 and EN-1997. Both E and C contributions peaked in December 2015, with a strong decay from January to June 2016.

Analysis of spatial patterns shows that the reduced EN signal in JAS 1982 can be explained in part by the delayed eastern Pacific warming (Supplementary Figs S7a,c–S8a). On the other hand, the EN contribution to the OND warming was similar in all three events (Supplementary Fig. S8b,d,f) but the warming trend had an important effect both in JAS and OND (Supplementary Fig. S9), making 2015 the warmest and 1982 the coolest of the three events.

### Drought severity

**Figure 2:**
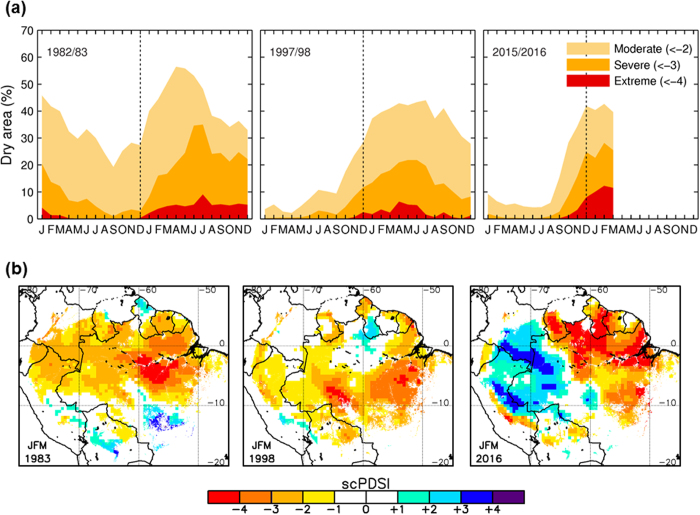
Drought severity over the Amazon rainforests during the three strong EN events. (**a**) Monthly time series of percentage of the area of the Amazonian rainforests affected by moderate, severe and extreme drought as indicated by the scPDSI. From left to right, results are presented for the three EN events in 1982–1983, 1997–1998, and 2015–2016. (**b**) Spatial patterns of scPDSI for the JFM season in 1983, 1998, and 2016. Data visualisations produced using IDL v8 (Exelis Visual Information Solutions, Boulder, Colorado).

2015 saw record-breaking warming in Amazonia. However, did this anomalous warming translate into enhanced drought conditions? The scPDSI suggests that up to a third of the Amazon rainforest is typically affected by moderate (scPDSI < –2) to severe (scPDSI < –3) drought conditions during strong EN years (Fig. 2). The extremely hot 2015/2016 event stands out by having the most extensive area under extreme drought severity (scPDSI < –4), with up to 13% of the rainforests undergoing extreme drought in February-March 2016 (Fig. 2a). This is up to a fifth more extreme drought area than in previous events, where such an intense drought severity did not affect more than 8-10% of the rainforests.

The spatial distribution of drought in 2015/2016 departed from the typical pattern seen in previous major EN events when drought was less severe but more widespread over Amazonia (Fig. 2b). This time, there was an unusual wet-dry dipole between southwestern and northeastern Amazonia. As a result, the mostly extreme drought conditions concentrated into a smaller region in the northeast. The moisture contrast is not a model artefact as it is also evident in precipitation anomalies of independent precipitation datasets based on satellite and *in-situ* observations (Supplementary Fig. S10). A similar wet-dry dipole was observed during EN 2009/2010 (Supplementary Fig. S11). Because of the strong contribution of the C index in EN 2009 and 2015, this wet/dry contrast can be attributed in large part to the different precipitation patterns resulting from El Niño warming in the central Pacific (2009 and 2015) and the eastern Pacific (1982 and 1997).

## Discussion

The year 2015 surpassed any warming anomaly observed in the last decade that has seen ‘one in a century’ extreme events (e.g., droughts of 2005 and 2010). Analysis of long-term temperature records suggests that 2015 is likely the hottest year over Amazonia in the last century (Supplementary Fig. S12) due to the combined effects of the El Niño conditions and the regional warming trend. The extreme heat coincided with an increased extent of extreme drought severity (scPDSI < −4) – a drought magnitude that occurs only in 2% of the months since January 1979.

Warming can further enhance drought severity from precipitation deficit through an increase in potential evapotranspiration (PET)^19^. As expected, PET was anomalously high during the period with record warming in late 2015 (Supplementary Fig. S15). To quantify the impact of warming on drought severity we compared the drought areas resulting from the scPDSI computed using varying PET (includes effect of warming) and PET set to climatological values (no effect of warming). This suggests that warming accounts for about a fifth of the anomalously high area under extreme drought severity observed during 2015/2016 (Supplementary Fig. S15). By March 2016, the effect of temperature on drought severity led to an additional 2.6% of the rainforests under extreme drought severity. For less intense drought severities, the effect of warming was almost negligible. This is consistent with the fact that the regions affected by extreme drought in eastern Amazonia coincided with those experiencing record warming (Figs 1b and 2b). Further work that considers the surface energy budget and land surface feedbacks^21^ would help to better quantify the impact of the record-breaking warming on drought across the basin.

Currently, ENSO neutral conditions are present, indicating that the warming phase is over. Different forecasts predict a transition to La Niña conditions during the boreal fall and winter 2016–2017 with varying probabilities (75% to 60%)^22^,^23^, although current conditions for equatorial heat discharge and easterly winds may suggest a lower probability for La Niña to occur (∼50%). Like in the 1982/83 and 1997/98 EN events (Supplementary Fig. S13), the seasons following El Niño peak in 2016 may also experience a significant warming over Amazonia. Although LST products derived from remote sensing data should be interpreted with caution over cloudy tropical regions^24^, satellite-based temperature anomalies over Amazonia still show a widespread warming in AMJ-2016. Moreover, drought conditions were still developing by the end of our study period in March 2016, but should have peaked by May or June.

The impacts of the observed climate anomalies on ecosystem function are still uncertain and remain to be documented. A number of studies reported impacts of earlier extreme temperature and drought events on rainforest. Tree mortality and biomass growth decline were observed after the 2005 and 2010 droughts, with implications also for the persistence of the Amazon forest as a carbon sink^8^,^9^,^10^. These impacts may be attributed not only to water deficits^8^ but also to heat stress^9^, with anomalously high air temperatures playing a role as important as precipitation deficits^25^,^26^. Fires in forested areas have been suggested to be the major agent of forest transition under an increased drought frequency scenario^27^. It is also believed that hot and dry conditions during EN years turn the Amazon ecosystem into a net carbon source^28^. However, it is has also been suggested that this only occurs during El Niño events with prevailing EP anomalies, whereas the Amazon rainforest remains a carbon sink during El Niño events with major contributions from CP anomalies^13^. Under continued global warming and a projected increase in the frequency of ENSO events^29^ more frequent record-breaking climate extremes are expected to occur in Amazonia during the coming decades.

## Methods

### Study regions

The Amazon forest was delimited by using the Combined Terra/Aqua yearly Land Cover product (MCD12C1)^30^. For this purpose, we selected pixels classified as “Evergreen Broadleaf Forest – EBF” within an area delimited by a geographical vector constructed from the political borders including South American provinces around the Amazonian forests. SST anomalies were extracted for the following sea regions: regions for El Niño 1 + 2 (EN12; 90°W–80°W, 0°–10°S), El Niño 3.4 (EN34; 120°W–170°W, 5°S–5°N), Tropical North Atlantic (TNA; 15°W–57.5°W; 5.5°N–23.5°N), and Tropical South Atlantic (TSA; 10°E–30°W, 0°–20°S). The analysis for the TNA and TSA regions was performed in terms of the gradient between the two regions (TNA-TSA). These study regions were also considered in other works^1^. The study regions are presented in Supplementary Fig. S1. Additionally, we used the *E* and *C* indices^15^ that correspond to SST anomaly patterns representing eastern and central equatorial Pacific warming, respectively (updated in near-real time at http://www.met.igp.gob.pe/datos/EC.txt).

### Skin and surface air temperatures

We used in our analysis monthly means of skin temperature (including both sea and land surface temperatures) extracted from the ERA-Interim (ERA-I) project developed by the European Centre for Medium-Range Weather Forecasts (ECMWF) at 0.75° × 0.75° latitude longitude global spatial resolution^31^. ERA-I covers the period from January 1979 until present, with a delay on the data delivery of around 2 months.

In order to extend back the study period to 1900, we used ERA20C and HadCRUT4 datasets of monthly temperature. ERA20C is the first reanalysis of the 20^th^-century developed by the ECMWF at 1° × 1° latitude longitude global spatial resolution covering the period 1900–2010^32^. ERA20C monthly temperatures were calibrated against the ERA-I temperatures for the overlapping period (1979–2010), and ERA20C was combined to ERA-I to obtain the monthly anomalies of skin temperature from 1900 to 2015 (Supplementary Fig. S7a). HadCRUT4 dataset includes monthly surface temperature anomalies relative to a 1961–1990 reference period on a 5° grid. This dataset is a collaborative product of the Met Office Hadley Centre and the Climatic Research Unit (CRU) at the University of East Anglia^33^. Note that ERA20C/ERA-I provides skin temperature, whereas HadCRUT4 provides air temperature.

Surface temperature anomalies at higher spatial resolution than the reanalysis and climate datasets were extracted from the Moderate resolution Imaging Spectrometer (MODIS) products at 0.05° spatial resolution (Supplementary Fig. S14). MODIS data over the Amazon region was extracted from the Thermal Amazoni@ web-based interface^34^.

Monthly temperatures extracted from the different datasets were converted to temperature anomalies and also to standardized anomalies. In the case of the ERA-I dataset, the reference climatological period was 1980–2014. ERA20C anomalies were obtained from a reference period of 1961–1990 in order to be consistent with the reference period used in the HadCRUT4 dataset (temperature anomalies are directly provided in this dataset, so additional calculations are not required). Surface temperature anomalies derived from remote sensing data (MODIS) were calculated using a reference period of 2001–2014.

Standardized anomalies were computed from the ratio between anomalies and the standard deviation for the reference period. Maps of standardized anomalies (e.g. Supplementary Figs S10b and S14) were categorized into 3 levels of significance: values between 0 and 1 are considered not significant, values between 1 and 1.96 are related to a probability of being anomalous between 1 and 2 sigma (68% to 95%), and values higher than 1.96 indicate a probability of being anomalous higher than 2-sigma (>95%).

### Self-calibrating Palmer Drought Severity Index

Monthly surface moisture variability over the period 1979–2016 was characterised using the Self-calibrating Palmer Drought Severity Index (scPDSI)^35^, which is a relative drought index that describes the severity of droughts and pluvials by comparison with the variations experienced during a reference period. The standardized moisture anomalies are derived from a basic soil moisture budget for the rooting zone that accounts for local soil water holding capacity, precipitation (moisture supply) and actual and potential evapotranspiration (moisture demand). The resulting index correlates well with variations in soil moisture, terrestrial water storage and runoff^19^,^36^. We computed the scPDSI for the Amazon basin at a spatial resolution of 0.5° using monthly precipitation and potential evapotranspiration (PET) from ERA-I reanalysis, and soil water holding capacity data from the Food and Agriculture Organization digital soil map of the world^37^. The calibration of the index was done over the reference period 1979–2015.

Monthly precipitation was derived from monthly means of daily forecast accumulations (MDFA) data. The MDFA are produced by averaging twice daily forecasts (00 and 12 UTC) over the month for forecast ranges of 0–12 hours, 12–24 hours and 24–36 hours^31^. For this work, drought severity was computed independently using the three different forecast ranges and then an ensemble average was produced. Monthly PET was computed using the Penman-Monteith method^34^ from monthly fields of surface net radiation, specific humidity, air temperature, wind velocity, and surface pressure data.

### Precipitation

Three precipitation datasets based on station and/or satellite records were used to evaluate the reliability of ERA-I precipitation estimates (Supplementary Fig. S10). This includes the 3B43 (V7) product from the Tropical Rainfall Measuring Mission (TRMM) Multisatellite Precipitation Analysis (TMPA)^38^, Global Precipitation Climatology Project (GPCP) dataset^39^, and the station-based Global Surface Summary of the Day (GSOD) product from the National Climate Data Center. Products were intercompared for the period 1998–2015 (Supplementary Fig. S10a).

## Additional Information

**How to cite this article**: Jiménez-Muñoz, J. C. *et al.* Record-breaking warming and extreme drought in the Amazon rainforest during the course of El Niño 2015–2016. *Sci. Rep.* **6**, 33130; doi: 10.1038/srep33130 (2016).

## Supplementary Material

Supplementary Information
