# Supplementary material for: Record-breaking warming and extreme drought in the Amazon rainforest during the course of El Niño 2015–2016
Source: Sci Rep. 2016 Sep 8;6:33130. doi: 10.1038/srep33130 (PMC5015046; doi:10.1038/srep33130)
Supplement: Supplementary Information [file srep33130-s1.doc]

**Record-breaking warming and extreme drought in the Amazon rainforest during the course of El Niño 2015-2016**

*Juan C. Jiménez-Muñoz, Cristian Mattar, Jonathan Barichivich, Andrés Santamaría-Artigas, Ken Takahashi, Yadvinder Malhi, José A. Sobrino, Gerard van der Schrier*

**Supplementary Figures**


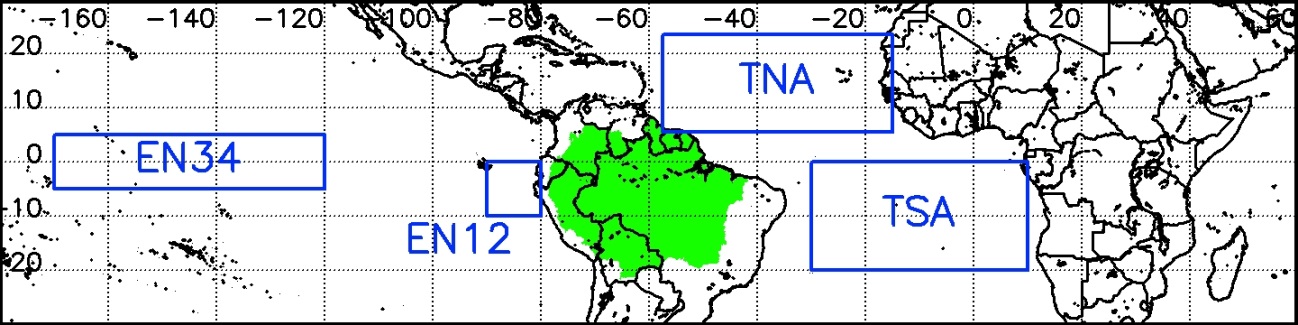


**Figure S1.** Sea regions (blue boxes) and delimitation of the Amazon forest (green area). EN34: El Niño 3.4; EN12: El Niño 1-2; TNA: Tropical North Atlantic; TSA: Tropical South Atlantic. Data visualisations produced using IDL v8 (Exelis Visual Information Solutions, Boulder, Colorado).


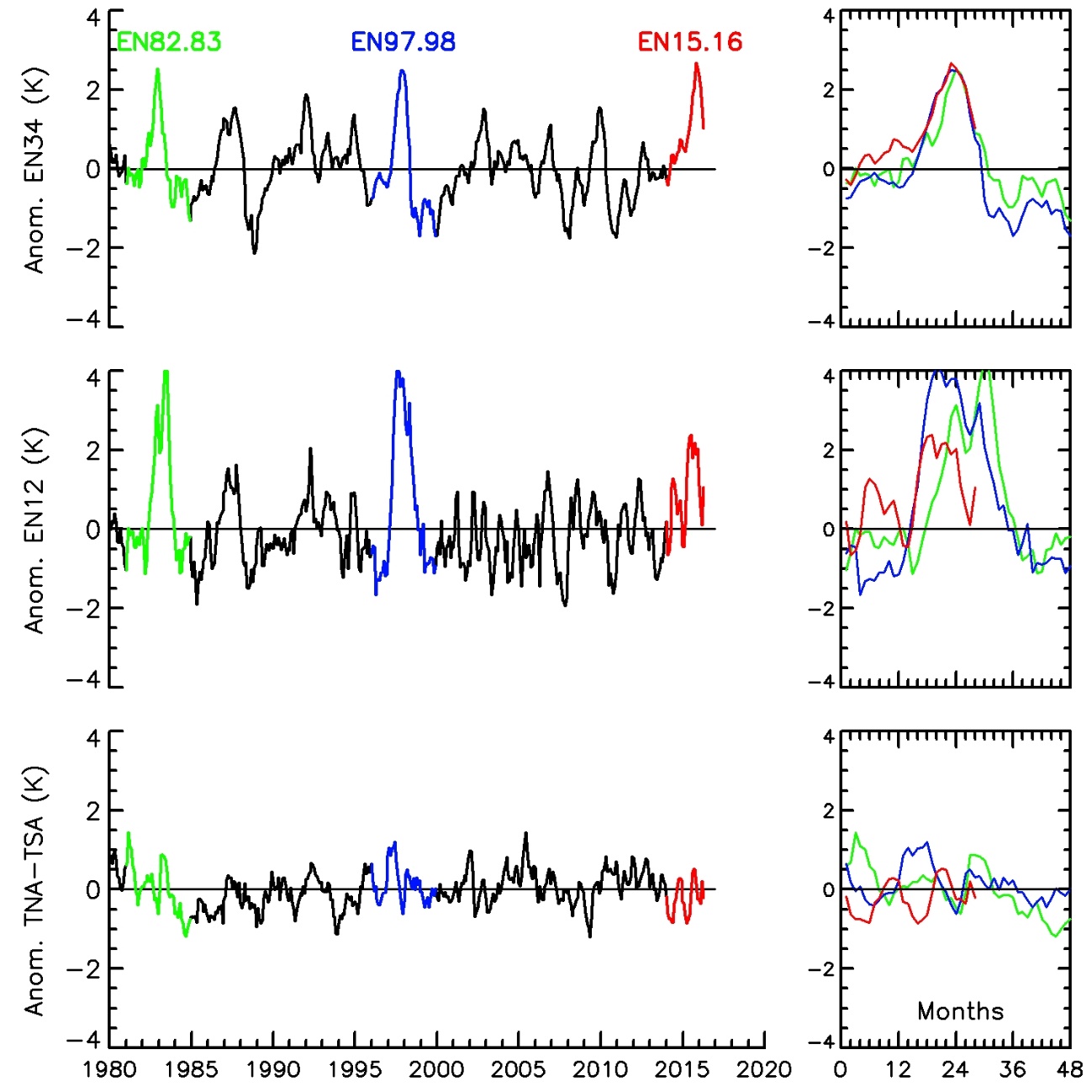


**Figure S2**. Monthly SST anomalies over EN34, EN12, TNA, and TSA regions for the 1979-2016 period. Anomalies over TNA and TSA regions are represented through the gradient between the two regions (TNA minus TSA). Green, Blue and Red colors refer to EN 1982/83, 1997/98 and 2015/16 events. Graphs on the right show an intercomparison between the three EN events during a four years period in the case of EN 1982/83 and 1997/98 (1981-1984, 1996-1999, respectively), and around three years period in the case of EN2015 (January 2014-April 2016).

**
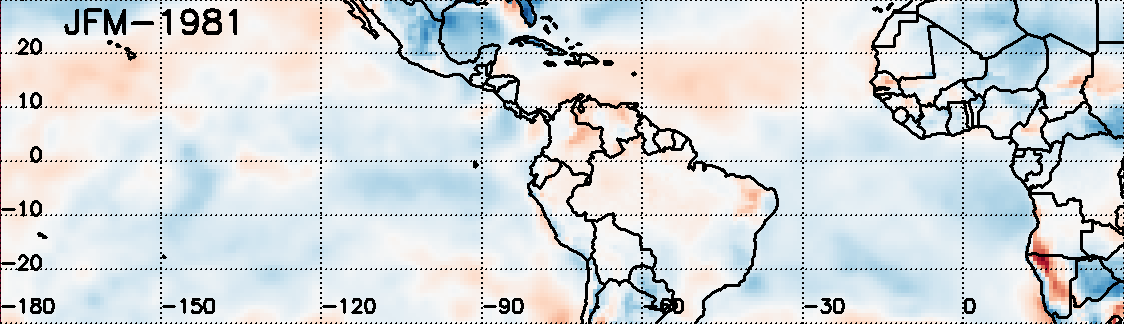
**

**
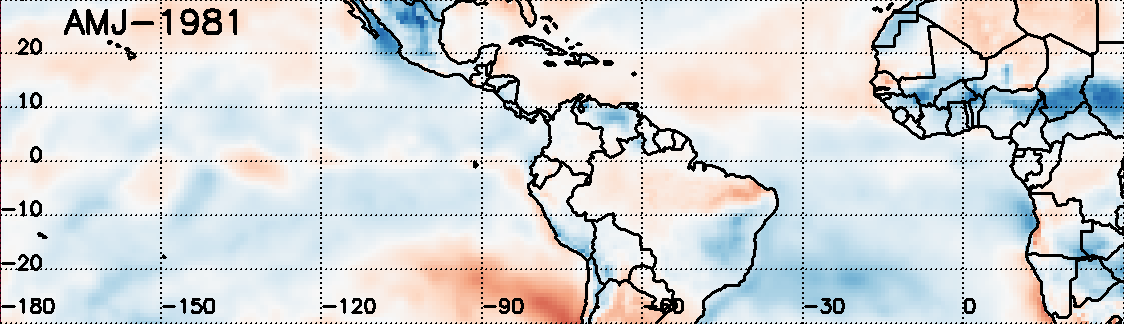
**

**
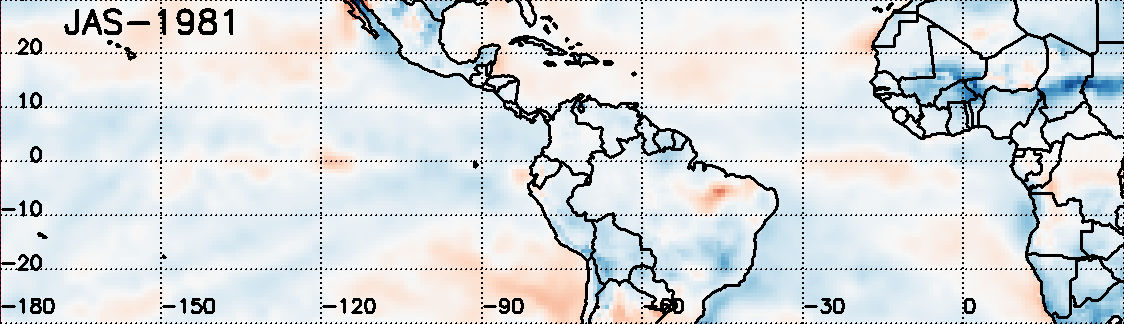
**

**
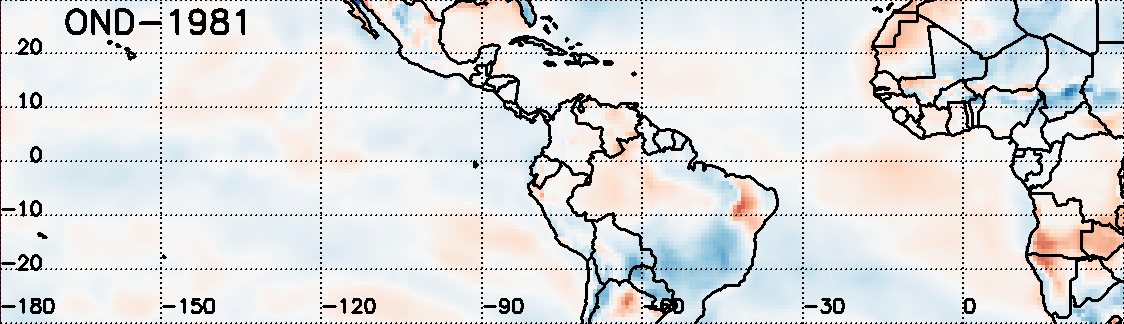
**


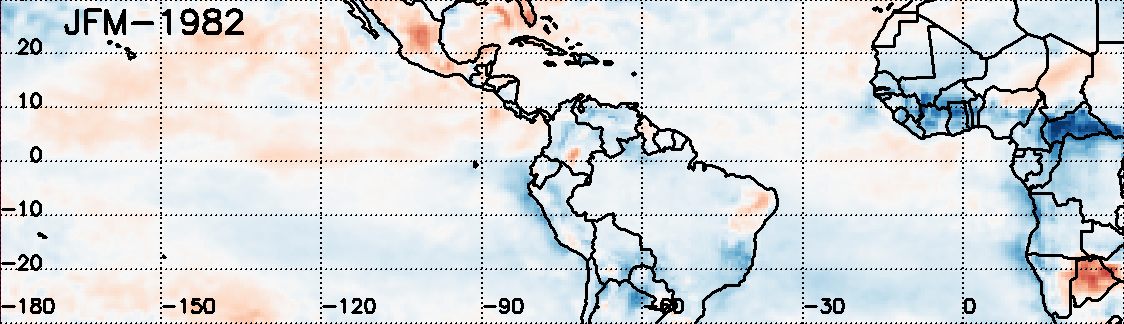


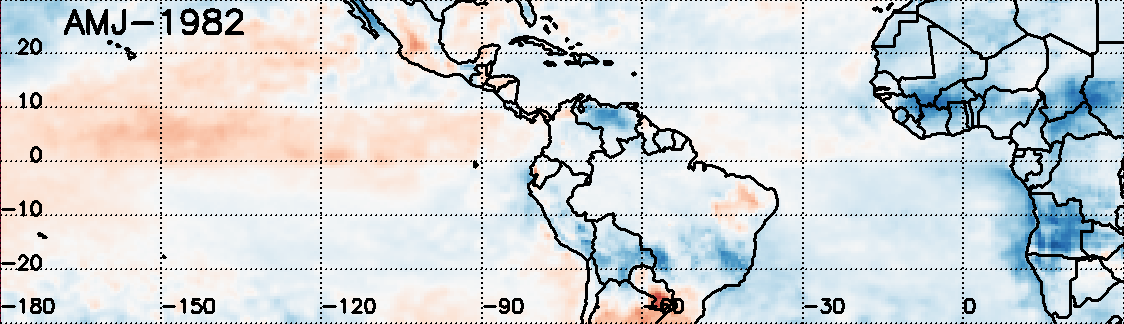


**
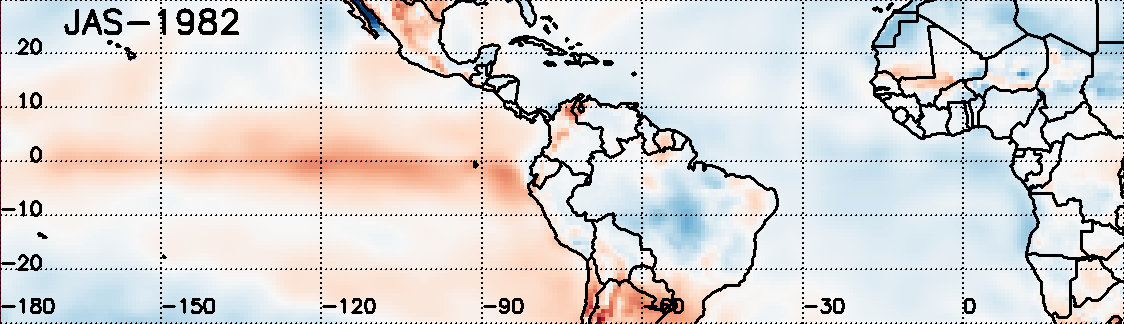
**

**
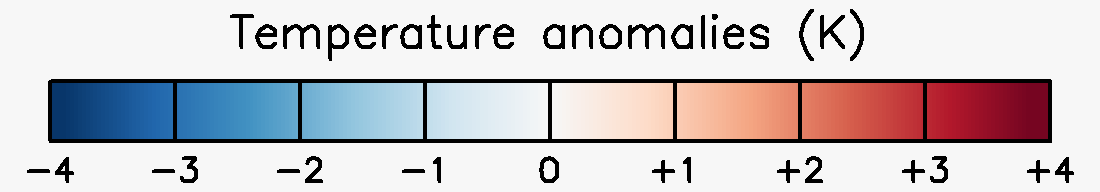
**

**Figure S3.** Surface temperature anomalies during the previous seasons to the development of EN event in late 1982. Data visualisations produced using IDL v8 (Exelis Visual Information Solutions, Boulder, Colorado).

**
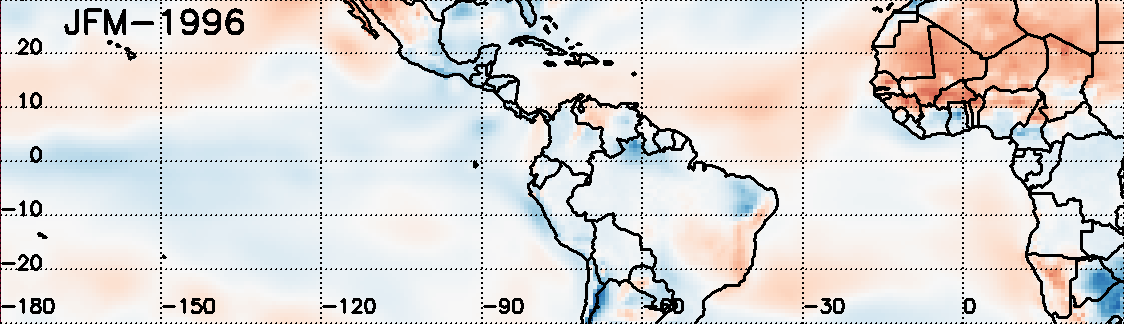
**

**
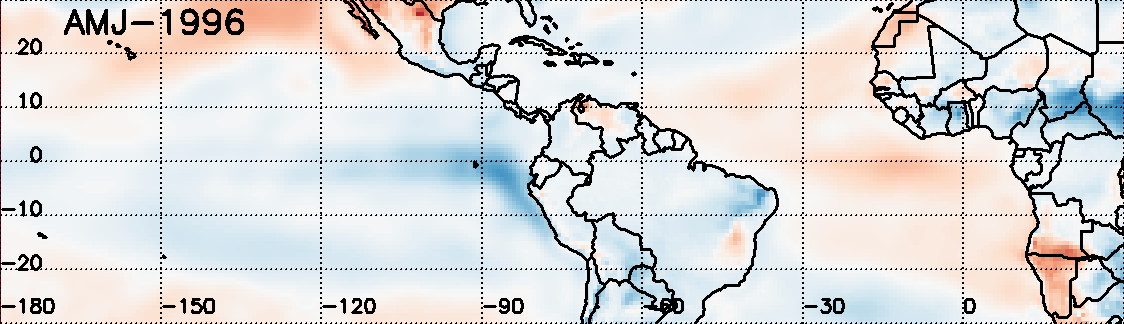

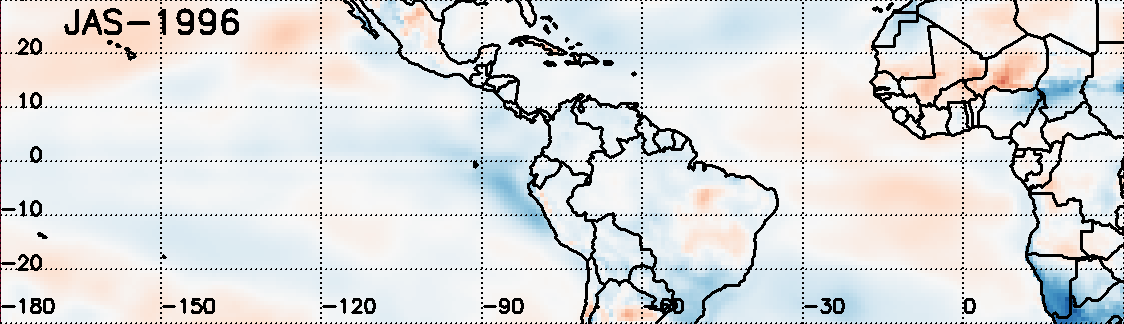

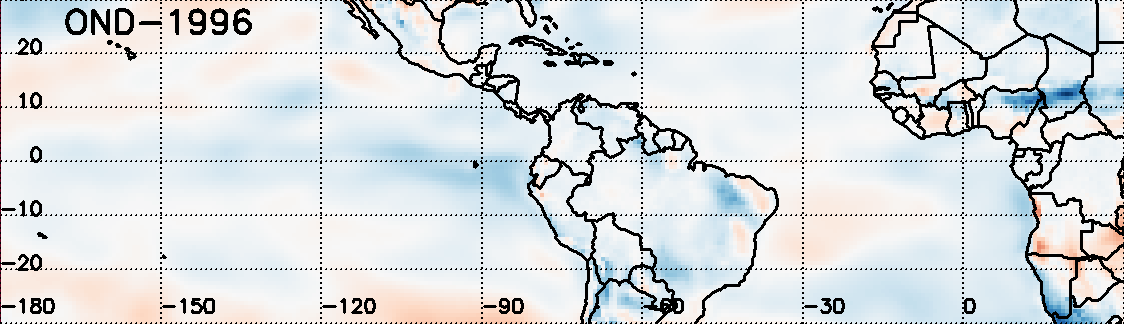
**

**
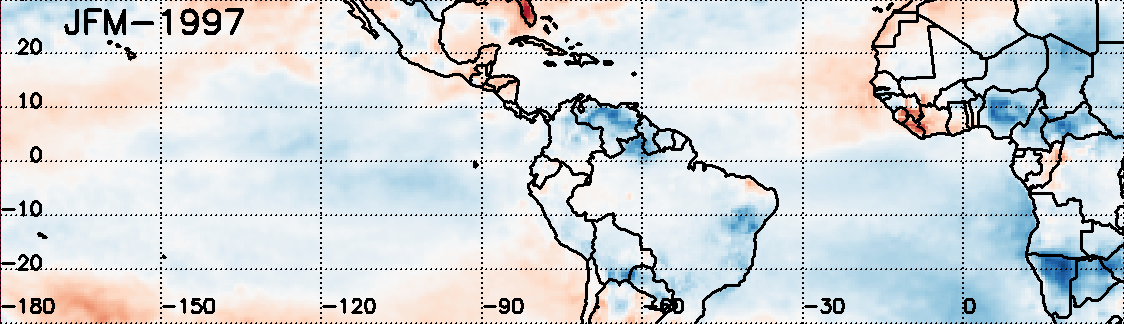
**

**
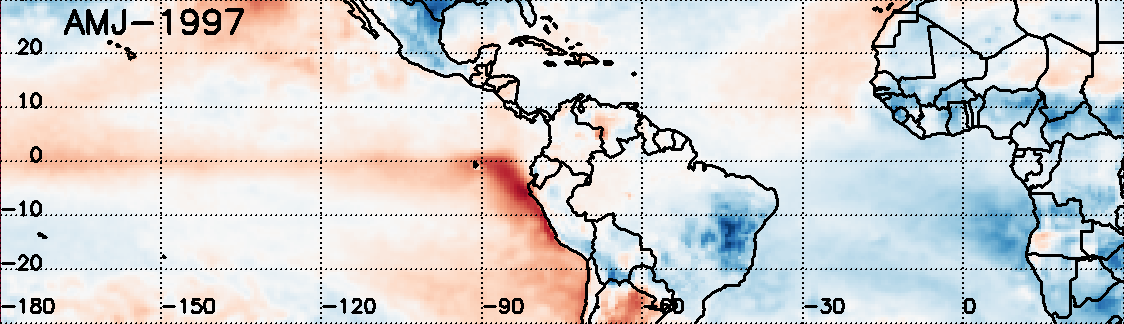
**

**
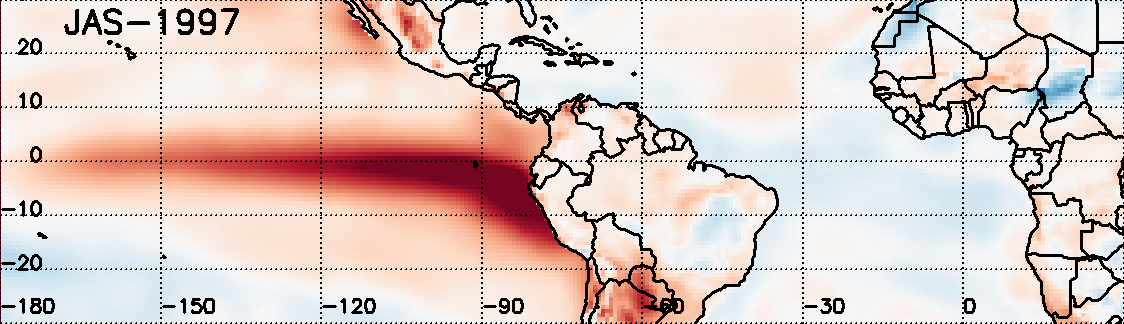
**

**
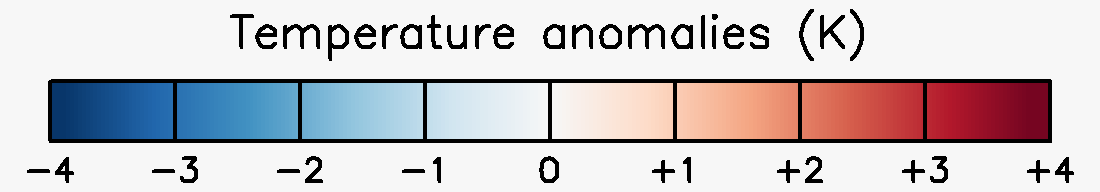
**

**Figure S4.** Surface temperature anomalies during the previous seasons to the development of EN event in late 1997. Data visualisations produced using IDL v8 (Exelis Visual Information Solutions, Boulder, Colorado).

**
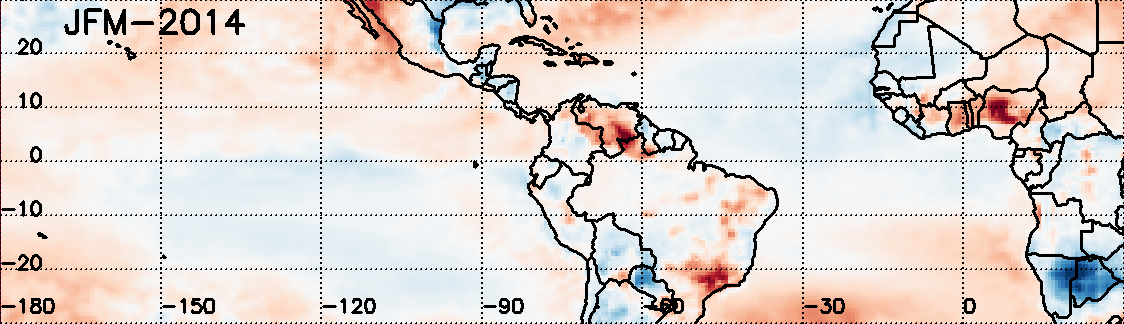
**

**
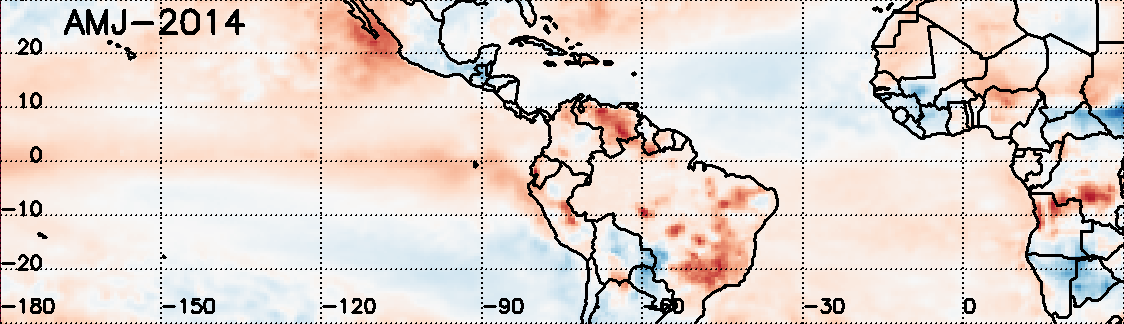
**

**
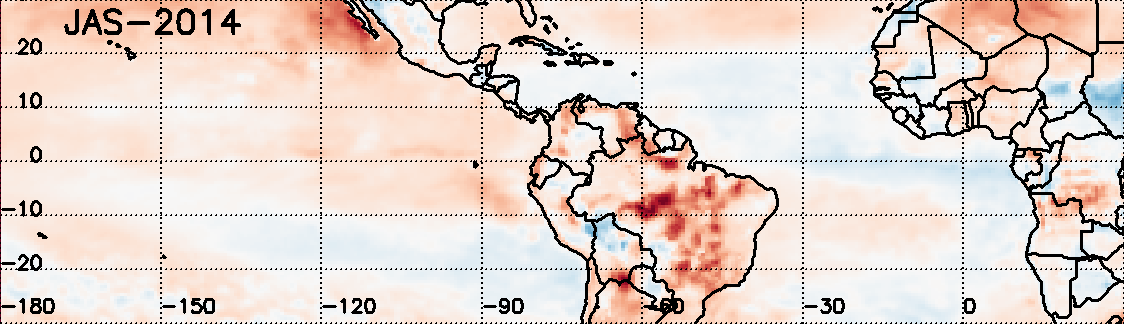
**

**
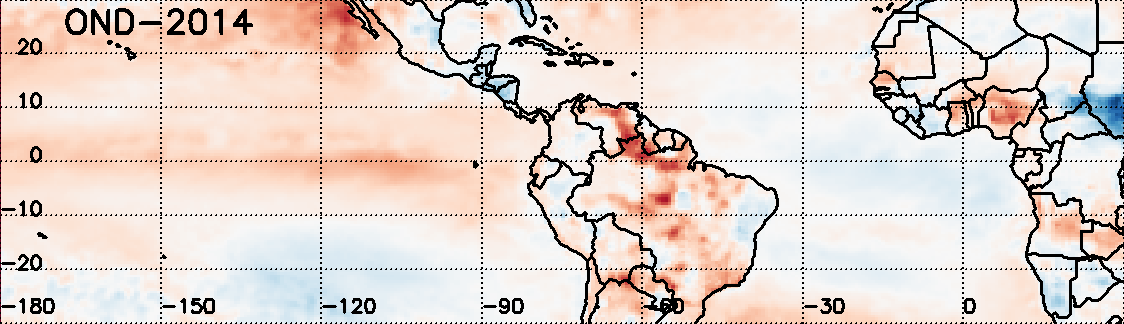
**

**
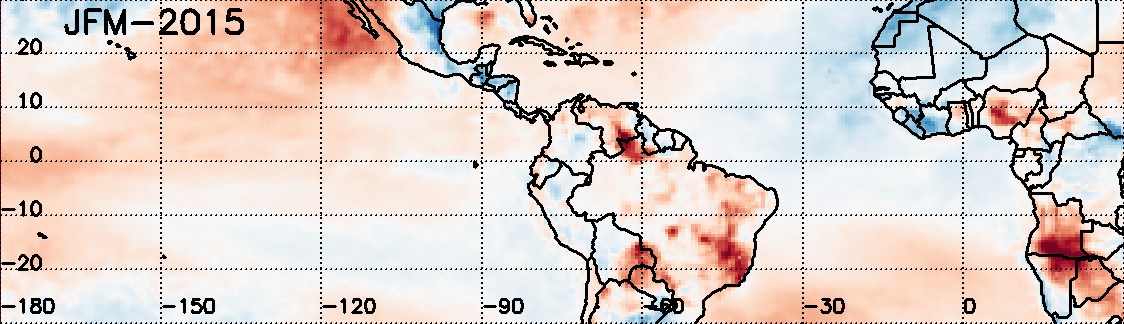
**

**
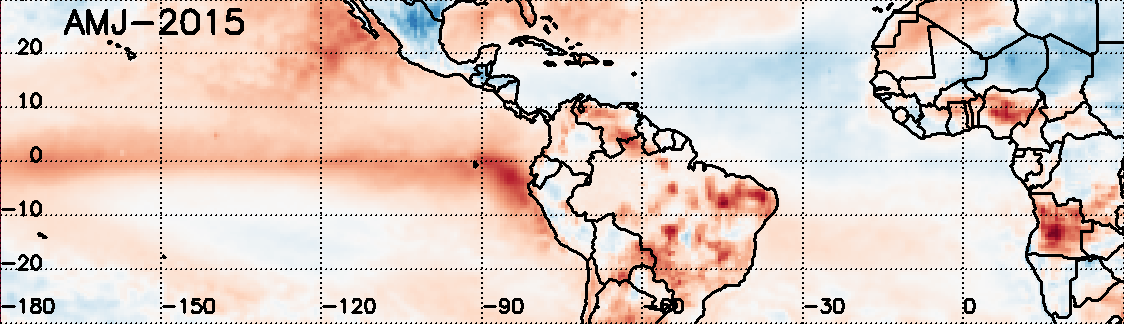
**

**
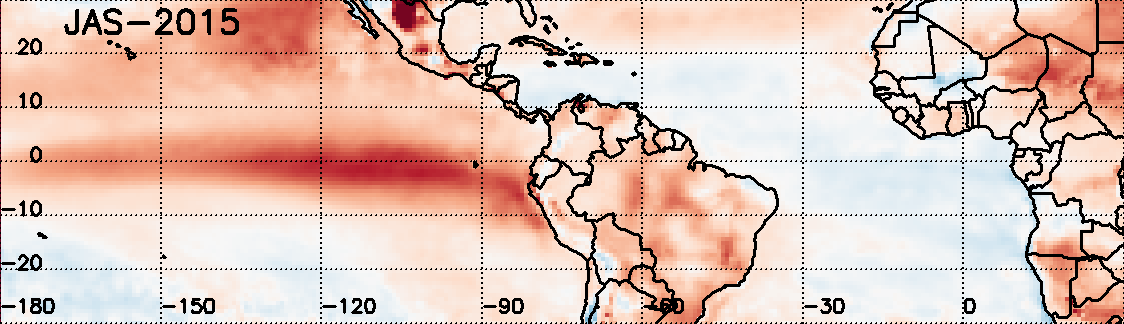
**

**
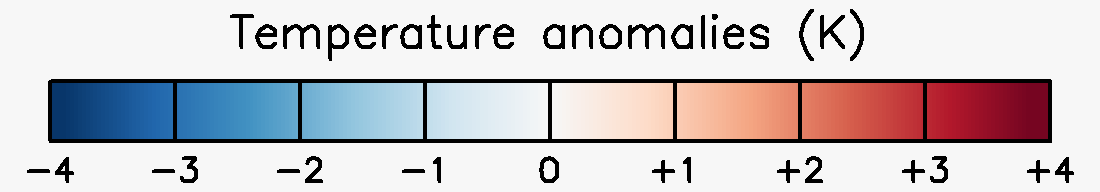
**

**Figure S5.** Surface temperature anomalies during the previous seasons to the development of EN event in late 2015. Data visualisations produced using IDL v8 (Exelis Visual Information Solutions, Boulder, Colorado).

**Figure S6.** Monthly series of E and C indices that correspond to SST anomaly patterns representing eastern and central equatorial Pacific warming, respectively (availabe at <http://www.met.igp.gob.pe/datos/EC.txt>). Last update: June-2016.

**Figure S7.** Linear regression coefficient maps of skin temperature onto the seasonal E and C indices for the JAS and OND seasons. Data visualisations produced using Grid Analysis and Display System (GrADS) Version 2.0.2. Copyright (c) 1988-2011 by Brian Doty and the Institute for Global Environment and Society (IGES).

**Figure S8.** ENSO (E and C) component of skin temperature anomalies (1981-2010). Data visualisations produced using Grid Analysis and Display System (GrADS) Version 2.0.2. Copyright (c) 1988-2011 by Brian Doty and the Institute for Global Environment and Society (IGES).

**Figure S9.** Linear trend component of skin temperature anomalies (1979-2015). Data visualisations produced using Grid Analysis and Display System (GrADS) Version 2.0.2. Copyright (c) 1988-2011 by Brian Doty and the Institute for Global Environment and Society (IGES).


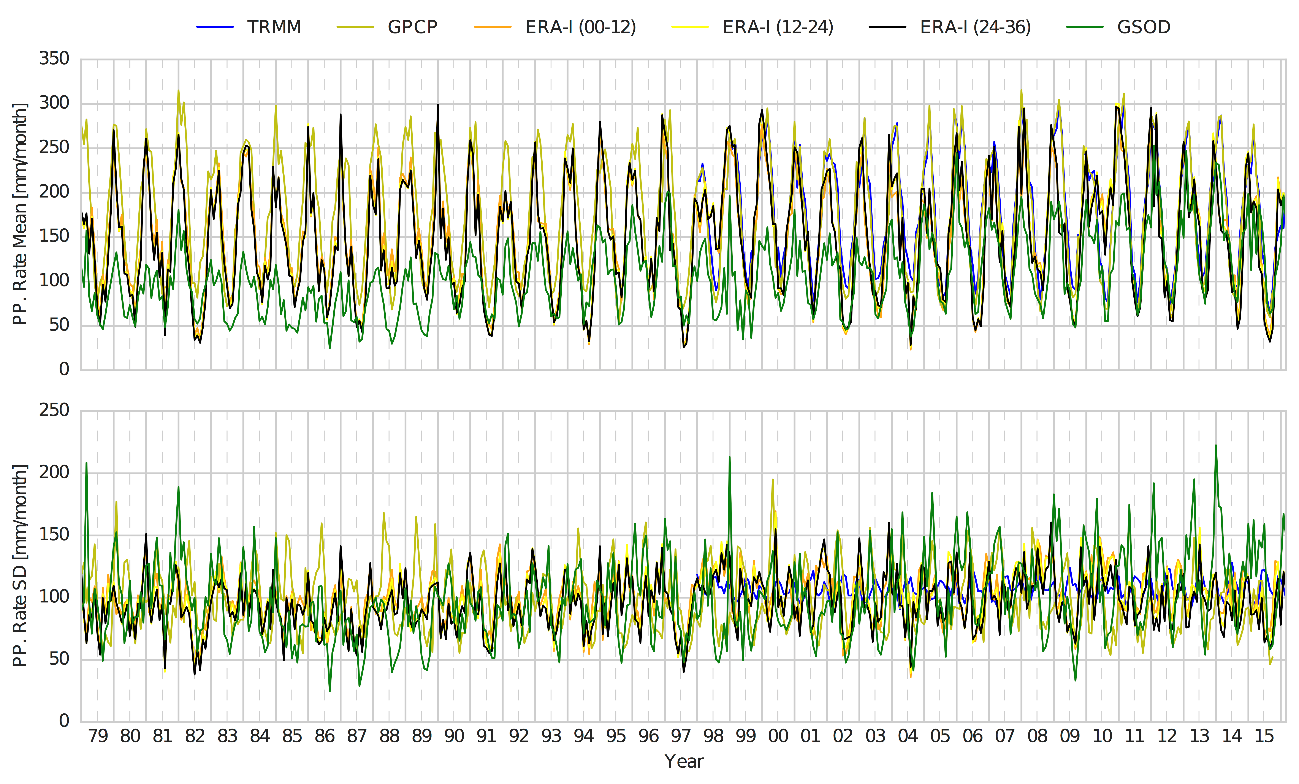


**(a)**

**TRMM GPCP**


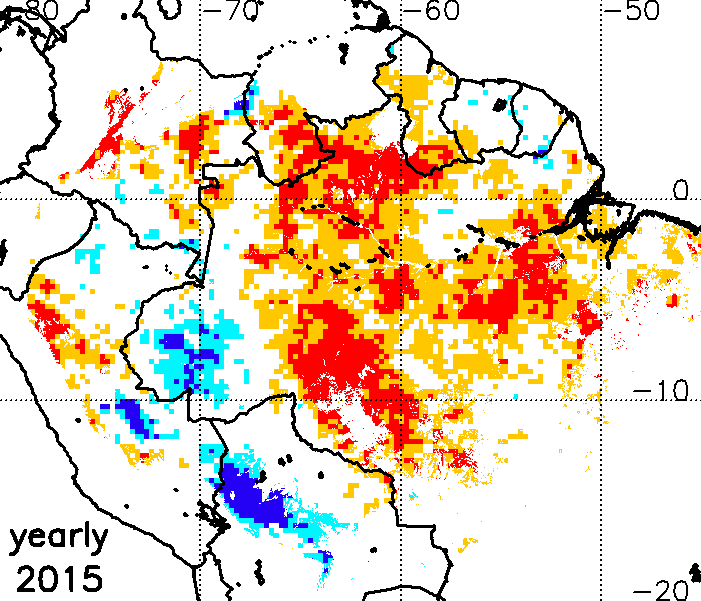

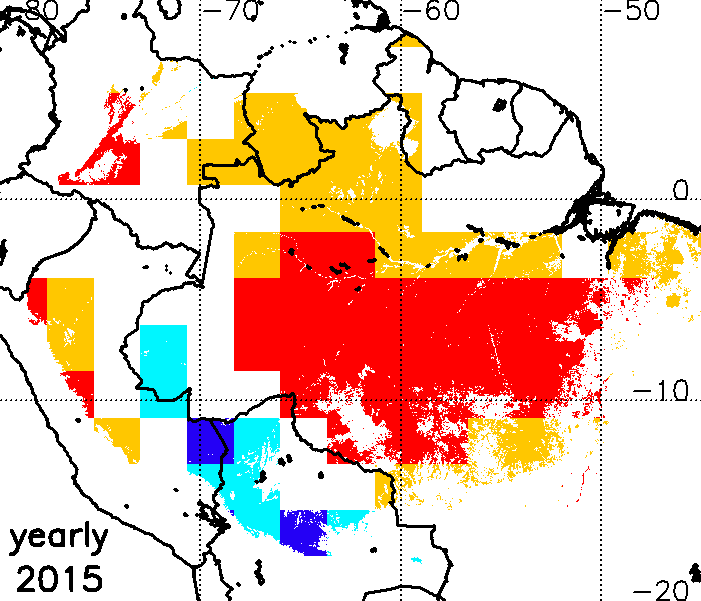


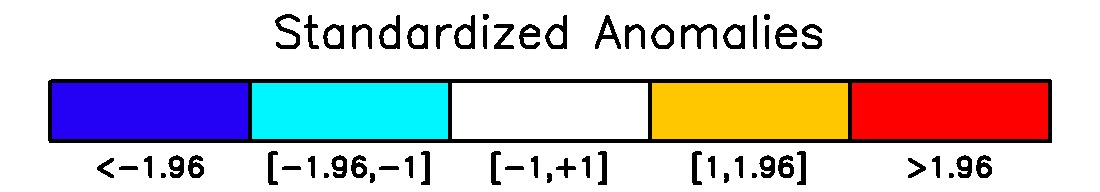


**(b)**

**Figure S10.** Intercomparison between monthly precipitation products extracted from different datasets. (a) Temporal evolution of monthly precipitation for the period 1998-2015 using different products. (b) Spatial pattern of yearly precipitation standardized anomalies over Amazonia using satellite-based data (TRMM) and observational data (GPCP). Data visualisations produced using IDL v8 (Exelis Visual Information Solutions, Boulder, Colorado).


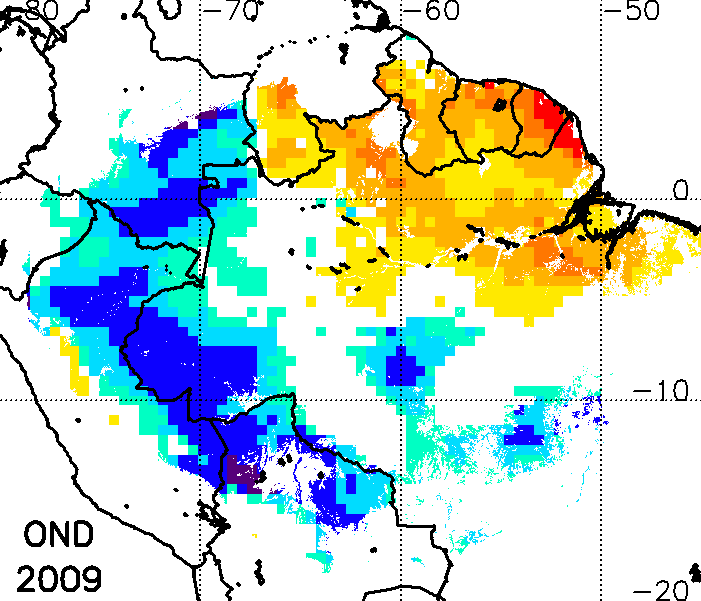

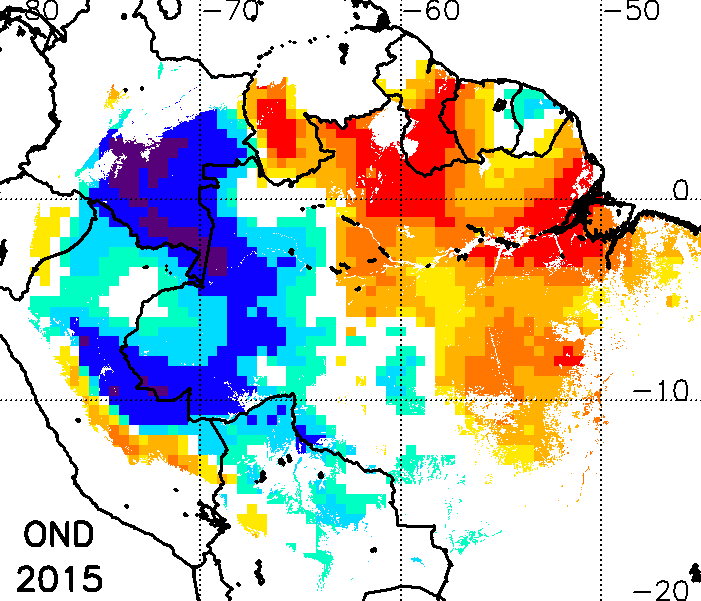


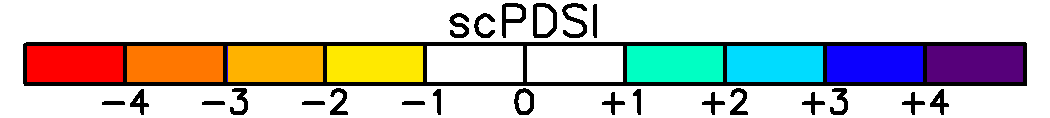


**Figure S11.** Self-calibrating Palmer Drought Severity Index (scPDSI) over the Amazon forest for the OND season during 2009 and 2015. Data visualisations produced using IDL v8 (Exelis Visual Information Solutions, Boulder, Colorado).


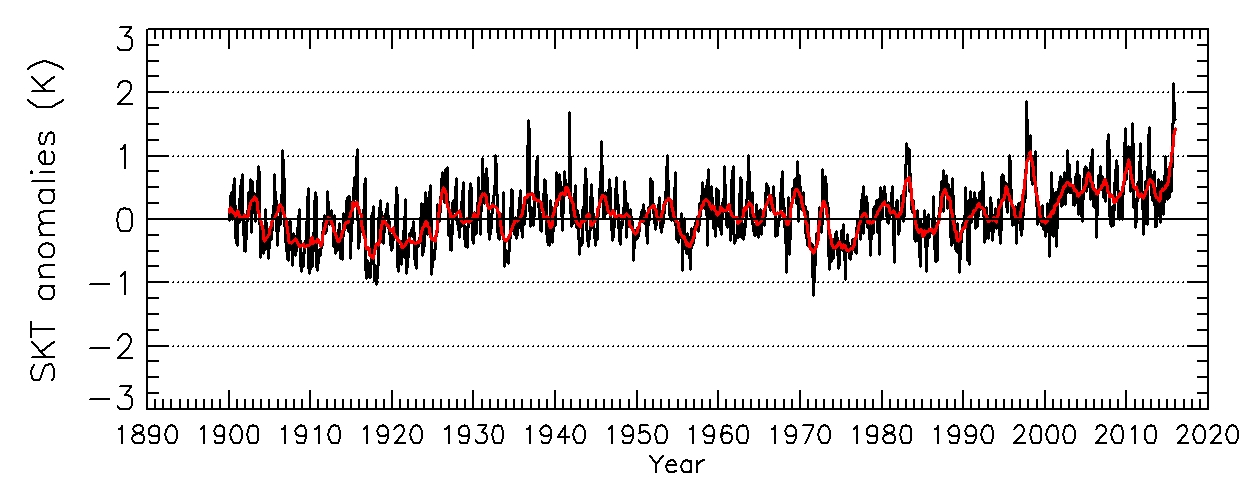


**(a)**


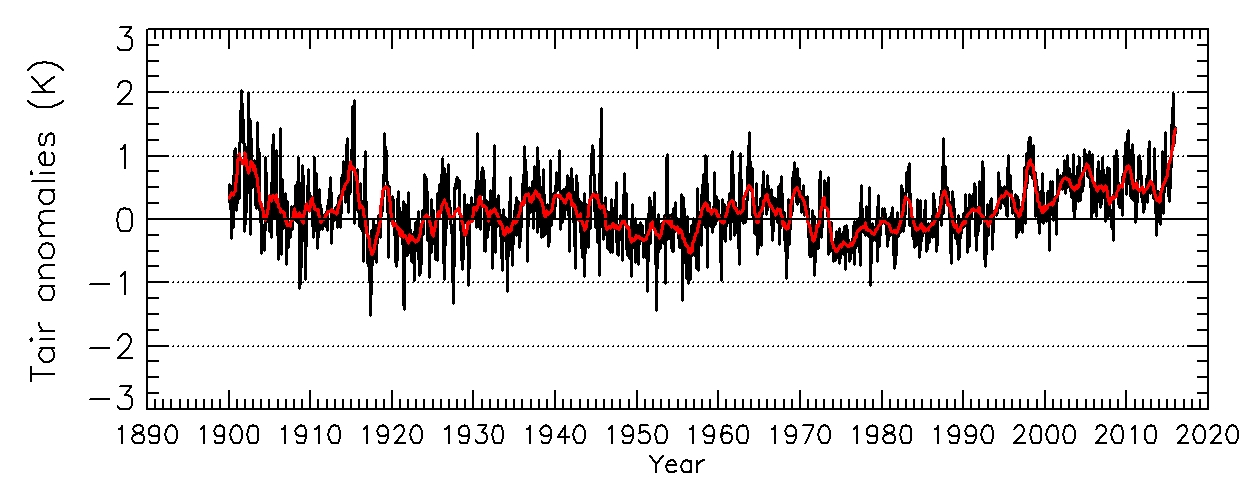


**(b)**

**Figure S12.** Time series of monthly temperature anomalies over the Amazon forest since 1900: **(a)** skin temperature anomalies extracted from a combination of ERA20C and ERA-Interim reanalysis products. **(b)** Air temperature anomalies extracted from the HadCrut4 climatic dataset. In both cases anomalies were computed using the reference period 1961-1990. Red line refers to the running mean for a period of 12 months.

| **1983** | **1998** |
| --- | --- |
| **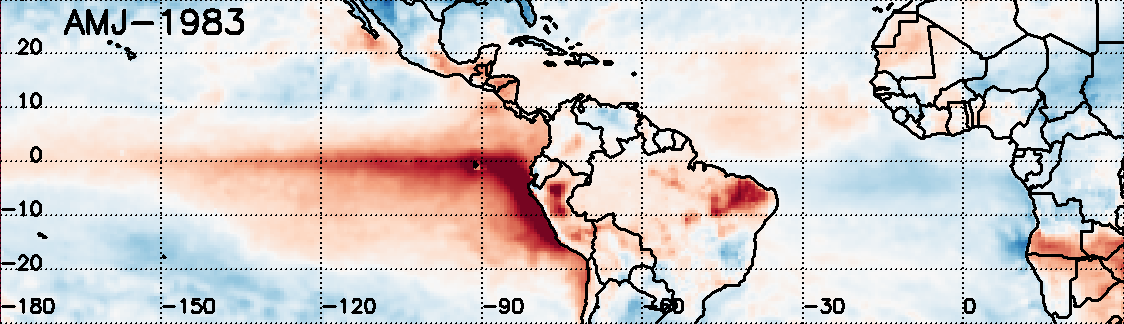** | **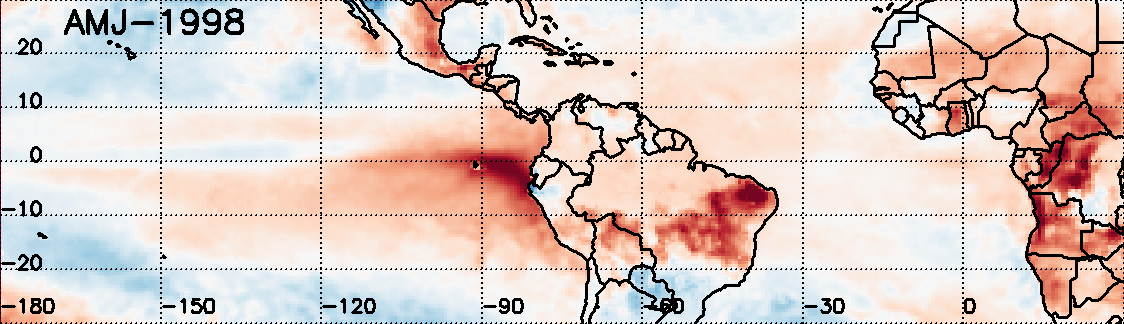** |
| **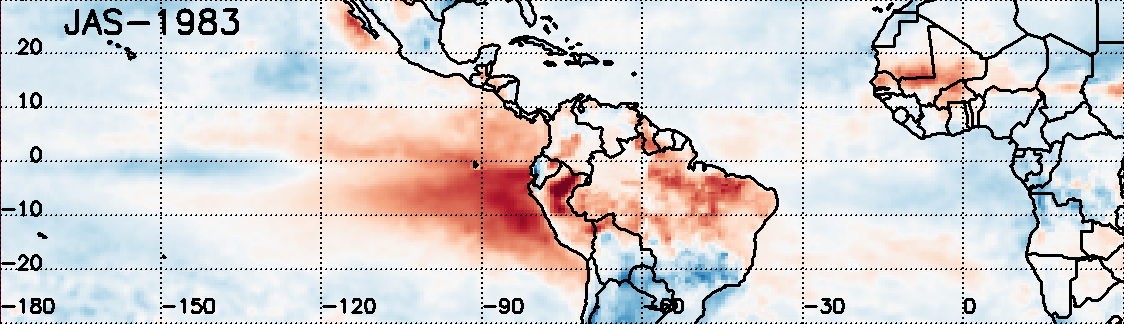** | **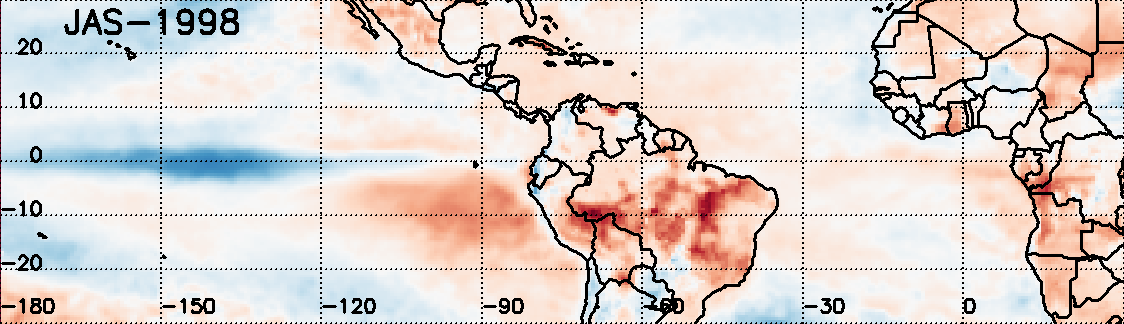** |
| **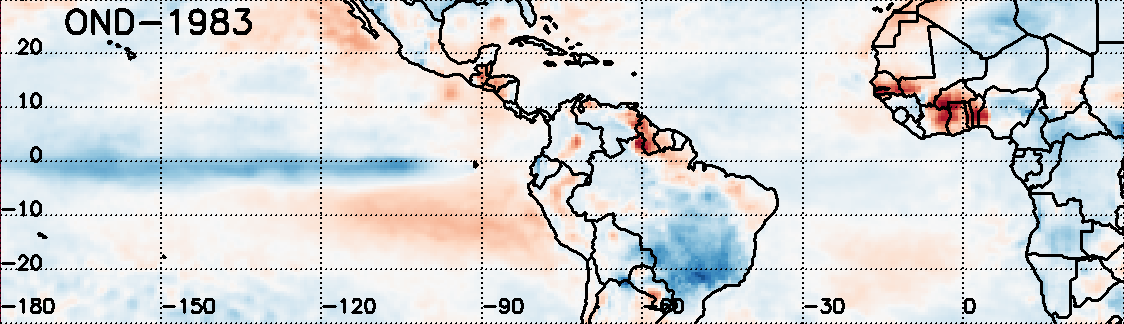** | **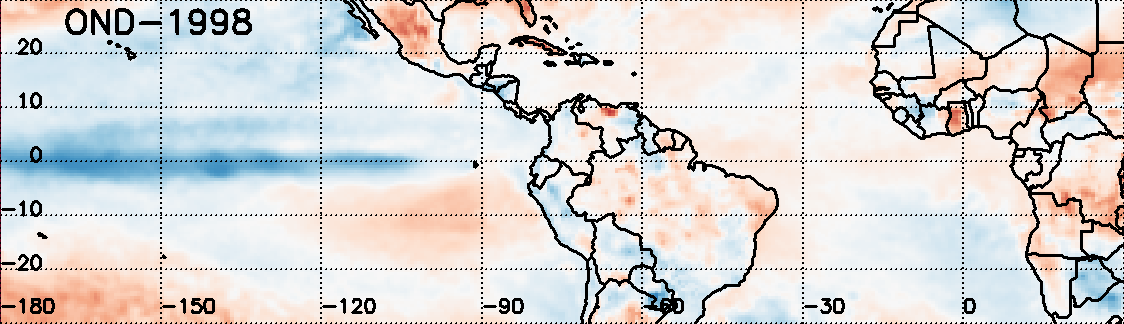** |

**
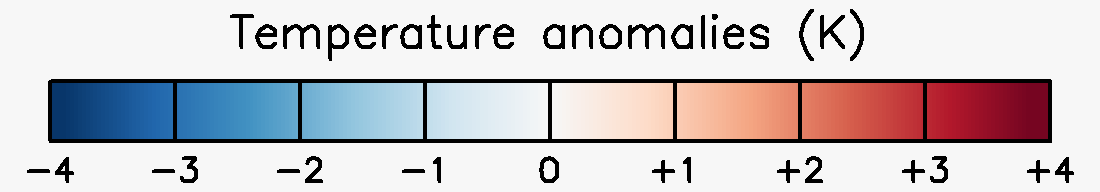
**

**Figure S13.** Surface temperature anomalies during 1983 and 1998. Data visualisations produced using IDL v8 (Exelis Visual Information Solutions, Boulder, Colorado).


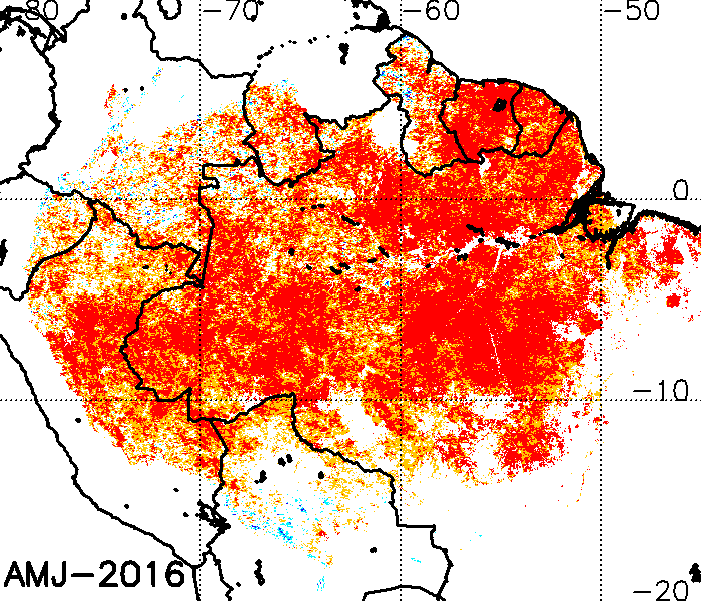


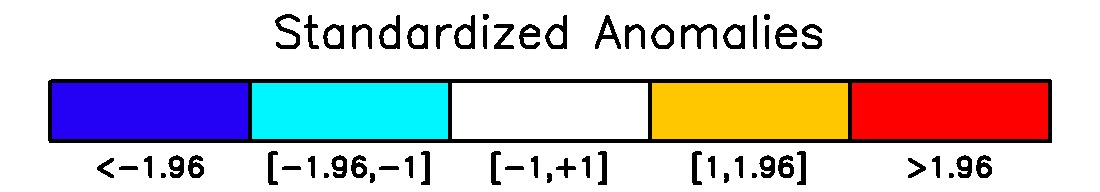


**Figure S14.** Land surface temperature standardized anomalies obtained from Terra-MODIS monthly LST products (MOD11C3 version 5) for the season AMJ-2016. Data visualisations produced using IDL v8 (Exelis Visual Information Solutions, Boulder, Colorado).


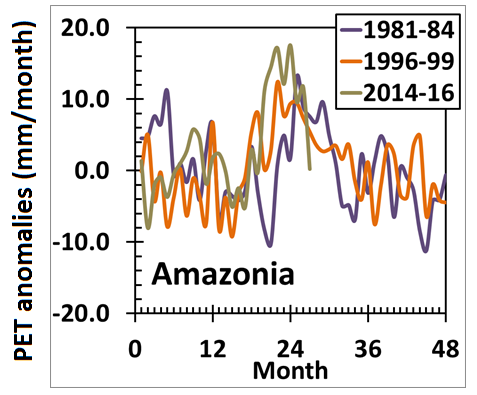


**(a)**


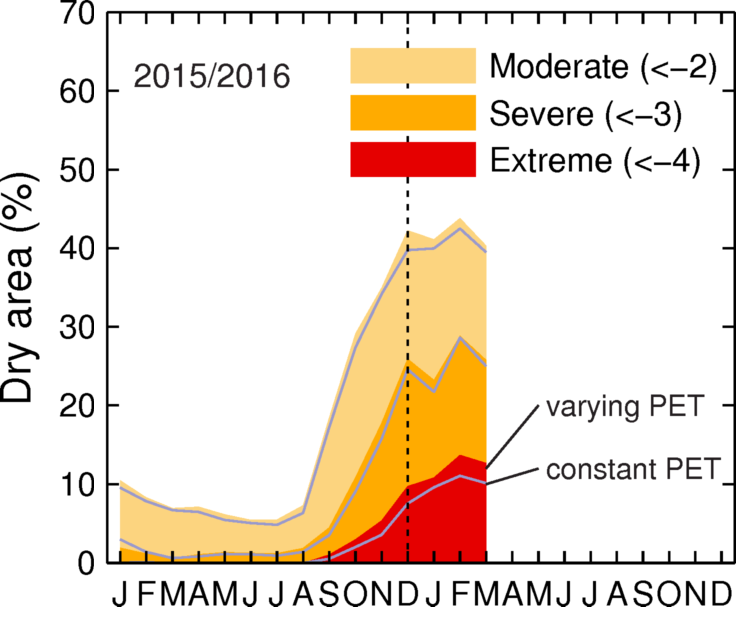


**(b)**

**Figure S15.** Contribution of the Potential Evapotranspiration (PET) term to the area affected by drought estimated with the scPDSI. **(a)** Evolution of PET anomalies during the course of the three EN events (1981-1984, 1996-1999, 2014-2016). **(b)** Differences between the dry area estimated with the scPDSI using a climatological mean value of PET (constant PET) and using the monthly value (varying PET). The grey line marks the dry area estimated from a constant PET.
